# Clinical prognostic indicators in multiple system atrophy

**DOI:** 10.1093/brain/awag160

**Published:** 2026-05-07

**Authors:** Yee Yen Goh, Viorica Chelban, Nirosen Vijiaratnam, Christine Girges, Manmohan Sandhu, Riona Fumi, Patrick W. Cullinane, Rahema Mohammad, Michele T. Hu, James B. Rowe, Nicola Pavese, Neil Archibald, Christopher Kobylecki, Thomas Foltynie, Huw R. Morris, Tom Warner, Zane Jaunmuktane, PROSPECT-M-UK, Henry Houlden

**Affiliations:** 1 Department of Neuromuscular Diseases, UCL Queen Square Institute of Neurology, UCL, Queen Square, London, WC1N 3BG, UK; 2 Department of Clinical and Movement Neurosciences, UCL Queen Square Institute of Neurology, UCL, Queen Square, London, WC1N 3BG, UK; 3 UCL Medical School, UCL, Gower Street, London, WC1E 6BT, UK; 4 Queen Square Brain Bank for Neurological Disorders, Queen Square Institute of Neurology, UCL, London, WC1N 3BG, UK; 5 Nuffield Department of Clinical Neurosciences, University of Oxford, Oxford, OX3 9DU, UK; 6 Medical Research Council Cognition and Brain Sciences Unit, University of Cambridge, Cambridge CB2 7EF, UK; 7 Clinical Aging Research Unit, Translational and Clinical Research Institute, Newcastle University, Newcastle upon Tyne, NE4 6BE, UK; 8 Department of Neurology, The James Cook University Hospital, South Tees NHS Foundation Trust, Middlesbrough, TS4 3BW, UK; 9 Division of Neuroscience, School of Biological Sciences, Manchester Academic Health Science Centre, University of Manchester, Manchester, M13 9NT, UK; 10 Manchester Centre for Clinical Neurosciences, Northern Care Alliance NHS Foundation Trust, Salford, M6 8HD, UK

**Keywords:** parkinsonism, survival, dysautonomia, neurodegeneration, progression, prognosis

## Abstract

Multiple system atrophy (MSA) is a neurodegenerative condition causing parkinsonism, cerebellar ataxia and/or dysautonomia. Typical survival is between 6-10 years, but some people die before five or after 15 years. This heterogeneity complicates advanced planning and clinical trial stratification. MSA prognostication studies have shown conflicting results, possibly due to diagnostic accuracy or study size.

We report results from a study of survival prognostic factors in a cohort of 555 MSA patients (including the largest post-mortem confirmed cohort to date of 254 people) gathered through the Queen Square Brain Bank and the PROSPECT-M-UK multi-centre prospective cohort study. Through PROSPECT-M-UK, 318 clinically diagnosed MSA patients (17 overlapped with the QSBB cohort) were followed up annually over 5 years. The QSBB cohort clinical data was collected through retrospective review of primary and secondary care documentation.

Survival analysis was performed using counting process Cox proportionate hazards modelling, Kaplan-Meier log-rank testing and landmark survival analysis to account for guarantee-time bias.

Mean onset age in the combined cohort was 58.7±9.0y with median survival of 8.25y (95% CI:7.88-8.63). 28.8% were clinically diagnosed in-life with MSA-P, 23.8% MSA-C, 40.2% mixed and the rest as non-MSA diagnoses. Later disease onset was associated with shorter survival (HR=1.04, *P*<0.001). The commonest cause of death was respiratory infection (67%) followed by disease related decline (20%). Median survival from indoor wheelchair use, gastrostomy insertion or development of unintelligible speech was consistently <1.5 years (95% CI upper limits<2.4 years), making these reliable late-stage disease markers. Using landmark analysis, at 3 years from onset, negative prognostic factors included recurrent falls, unintelligible speech, use of catheters and of medication for orthostatic hypotension (HR = 1.57, 3.29, 1.76, 3.29;all *P*<0.05). At 5 years from onset, mobility milestones including walking aid use, outdoor and indoor wheelchair use (HR = 1.70, 1.93, 2.62;all *P*<0.01) became significant, whilst dysautonomia milestones (catheter and orthostatic support medication use) were no longer significant. Median individual Unified Multiple System Atrophy Rating Scale (UMSARS) progression rate (n=91) was 10.27 (IQR:5.31-14.30) points/year and did not correlate with symptom duration. Higher baseline UMSARS and faster UMSARS progression were negative prognostic factors of survival from baseline review (HR=1.03 and 1.07 respectively, both *P*<0.001).

We show that in-clinic rating scales and clinical milestone assessment can aid MSA prognostication. Importantly, prognostic factors demonstrate time-dependent variability, which may contribute to previous heterogeneity observed in smaller studies. This knowledge is important for patient care and should inform future clinical trial stratification.

## Introduction

Multiple system atrophy (MSA) is a progressive neurodegenerative condition characterised by a combination of cerebellar, parkinsonian and autonomic features. Prognosis is poor, with a median survival of between 6.4 to 9.8 years.^1–8^

Survival in MSA is variable, with some patients deteriorating rapidly and passing away within 5 years, whilst there is a subset of patients who display a ‘benign’ version of symptoms, living more than 15 years from symptom onset.^9,10^

Accurate prognostication in MSA is important for several reasons. First, it is vital for clinical advance care planning, to ensure patients receive the best care possible. Second, with the advent of several disease modifying drugs in clinical trials for MSA, improved prognostication will allow better stratification for more meaningful results. Given the rarity of MSA (estimated prevalence of 1.9 to 3.3 cases per 100,000), trial sizes tend to be small, and at greater risk of being affected by this heterogeneity in survival.^11^ Lastly, understanding factors associated with faster or slower disease progression could lead to better understanding of underlying pathophysiology and potential treatment options.

Clinical prognostic indicators in MSA have previously been reported, suggesting a negative impact of later age of onset, female sex, early dysautonomia, early falls, type of symptom onset and clinical subtype^1–4,7,8,12–16^. However, results so far have been inconsistent between studies (see Supplementary Table 1). This may be related to study size and diagnostic accuracy given previous neuropathological case series showing a possible misdiagnosis rate of 20-38%.^17,18^

In this study, we analyse clinical prognostic indicators of survival in 254 post-mortem confirmed MSA patients from the Queen Square Brain Bank and 318 clinically diagnosed patients recruited prospectively through the PROSPECT-M-UK observational study.

Using this uniquely large clinical and pathological cohort, our key aims are to investigate: 1) the variability of life expectancy in people with MSA, 2) whether specific early clinical indicators such as disease severity milestones or validated rating scales can be used to predict survival in patients with MSA. In this study, we have specifically chosen to investigate clinical phenotypic indicators of prognosis, rather than laboratory-based biomarkers, in order to utilise synchronised matching data points across the different cohorts.

## Materials and methods

### Ethics

Ethical approval for the PROSPECT-M-UK study was granted by the Queen Square Research Ethic Committee (REC Reference: 14/LO/1575), whilst the Queen Square Brain Bank data collection and storage ethical approval was granted by the London – Central Research Ethics Committee (REC Reference: 23/LO/0044). Written consent in accordance with the Declaration of Helsinki was obtained for all participants.

### Participants and evaluation

#### PROSPECT-M-UK Cohort

The PROSPECT-M-UK study is an observational study of people with atypical parkinsonism, recruited across seven UK study sites, previously described elsewhere^19,20^.

For this analysis, PROSPECT-M-UK participants who fit either of the following criteria were included:

1. Participants clinically diagnosed by expert clinicians with probable MSA (using the 2008 Gilman criteria^21^) and no subsequent clinical or neuropathological diagnostic revision
2. Participants with a neuropathological diagnosis of MSA following post-mortem review, regardless of clinical diagnosis during life.

Baseline assessments for the clinical cohort were between 2 July 2015 and 2 October 2023, with a census date of 31 October 2023 for survival analysis. Longitudinal follow up was planned for 6 months, 12 months and then yearly until 60 months. Reporting of this study was performed in accordance with STROBE Guidelines (see Supplementary Materials for completed checklist).

Validated rating scales were performed via in-person assessment and participant self-evaluation questionnaires. Instruments applied included the Unified Multiple System Atrophy Rating Scale (UMSARS) and MSA Quality of Life Score (MSAQoL).

#### Queen Square Brain Bank (QSBB) Cohort

The Queen Square Brain Bank for Neurological Disorders specialises in parkinsonian movement disorders and collects donated brains and clinical data from across the UK. Neuropathological evaluation followed standard QSBB protocols with MSA diagnosis established using 2008 and later, 2022 consensus criteria^21,22^.

Clinical diagnosis was made by expert clinicians based in UK secondary or tertiary care facilities. Data was extracted from contemporaneous notes of patients seen at the National Hospital for Neurology and Neurosurgery, Queen Square, primary care documentation, secondary and tertiary care correspondence, QSBB neuropathology and clinical summary reports. Systematic clinical data extraction was performed using a pre-specified data extraction tool by two separate investigators. A random selection of 15 patient records was collected in duplicate to review for inter-investigator coding concordance, with >95% concordance and Cohen’s kappa of 0.9107.

Where performed, pathological subclassification was done by experienced neuropathologists, classifying cases as MSA with predominantly striatonigral degeneration (MSA-SND), predominantly olivopontocerebellar atrophy (MSA-OPCA) and MSA with equal SND and OPCA involvement (MSA-SND=OPCA).

##### Data quality and missing data

Investigators rated the quality of patient notes available on a semi-objective basis. High quality documentation included those with both contemporaneous primary and secondary care documentation (n=90). Mid-level quality documentation included those with only one of either primary or secondary care documentation (n=135), whilst low quality documentation was where only second-hand clinical summaries were available (n=29).

Imputation was not considered appropriate for missing data, due to concerns around data not missing at random.

##### Data definitions and collection methods

Disease onset was defined as the onset of first patient-reported motor or autonomic symptom (excluding constipation and REM sleep behaviour disorder) attributed to probable or possible MSA using the 2008 Gilman criteria. Autonomic symptoms included genito-urinary symptoms, erectile dysfunction and symptoms of orthostatic hypotension. No specific data was available on female sexual dysfunction, and to avoid bias, subsequent sensitivity analyses were performed, excluding erectile dysfunction as an onset symptom.

Recurrent falls were defined as >1 fall within 12 months, with onset documented as the date of the second fall. Catheter use was defined as irreversible need for catheter use, whether with intermittent self-catheterisation, urethral or suprapubic catheter. Unintelligible speech was defined either by self-reported assessment, speech therapy assessment suggesting >50% unintelligibility or the required use of communication aids.

For the PROSPECT cohort, patient-reported time of onset, time to diagnoses and disease milestones were collected during clinician review or using a self-completed patient history questionnaire at baseline and on follow-up visits. For the QSBB cohort, these were determined through retrospective note review. Date of death was determined using national registry and next-of-kin reported information.

Strict event timing principles were applied as follows: (1) Information from the assessment closest in time to event onset was prioritised, (2) A margin of error of no more than ±2 months was allowed (3) Clearly defined surrogate markers of onset were accepted e.g. defining start of intermittent catheterization as first recorded GP intermittent catheter prescription.

### Statistical analysis

Analysis was done using RStudio with R version 4.1.1 (2021-08-10). The *survival, survminer, car* and *survivalAnalysis* packages were used for survival analysis whilst *ggplot2* and *forest_model* were used for plot generation. GraphPad Prism 10 was also used for plot generation.

Survival from onset and point of landmark analyses were the main outcomes analysed for all patients. For patients recruited through PROSPECT-M-UK, survival from baseline review was also considered for some covariates.

Time independent variables (e.g. age at onset, clinical subtype) were examined using standard Cox proportional hazards analysis. Time varying variables (e.g. time to milestone attainment) were examined using a counting process dataset and landmark analysis to avoid guarantee time bias^23^.

Guarantee time bias occurs when a studied variable occurs after study enrollment. To experience the variable event, patients must survive until it occurs, creating a period of ‘immortality’ that affects survival analysis. As such, specific statistical analysis to account for this was performed.

Proportional hazards assumptions were tested using *coxzph* and Schoenfield graphical outputs. Influential observations and outliers were reviewed, and tests for multi-collinearity and variance influence factors were performed for all models. Where multiple testing was performed, *p* values were adjusted using the Bonferroni method.

The initial time point for landmark analysis was chosen based on median time from onset to diagnosis. Subsequent landmarks points were at 1-year intervals for another 2 years. Analysis was performed on cohort subsets of followed up participants known to be alive at each landmark point. Numbers of included participants at each landmark point are provided in Supplementary Table 1.

Kaplan-Meier log rank survival analysis was used for categorical variables (e.g presence of autonomic symptoms at onset, sex, levodopa response, early vs late milestone onset).

Normally distributed data (using the Shapiro-Wilk test) is reported as mean ± standard deviation (SD) whilst non-normally distributed data is reported as median and interquartile range (IQR). Grouped means were compared using ANOVA, unpaired T-tests or Kruskal-Wallis depending on group number and distribution. Where relevant, pairwise post-hoc comparison between group means was performed using the Tukey method.

## Results

### Descriptive demographics

#### Cohort Demographics

Table 1 provides an overview of both cohorts.

The longest surviving neuropathologically confirmed participant survived 17.97 years from onset. Although median survival from onset was similar between cohorts (*P* = 0.57), age of disease onset and death was later in the PROSPECT-M-UK cohort (*P* = 0.017 and *P* = 0.0051 respectively). Chi-square testing showed no difference in sex distribution between the cohorts (*P* = 0.102).

##### QSBB post-mortem cohort

14.9% of the QSBB cohort did not receive an MSA diagnosis in life, with the most common misdiagnosis being Parkinson’s Disease (PD) in 18 cases (7.1%), followed by progressive supranuclear palsy (PSP) with 12 cases (4.7%).

Cause of death was available in 144 (56.5%) of the post-mortem cohort. Of these, 66.7% (n = 96) were due to respiratory infections, whilst disease related decline was responsible for another 20.1% (n = 29). 4.2% deaths (n = 6) were due to non-infective cardiorespiratory failure, whilst another 2% (n=3) were related to unspecified sepsis.

##### PROSPECT-M-UK specific demographics

**Figure 1:**
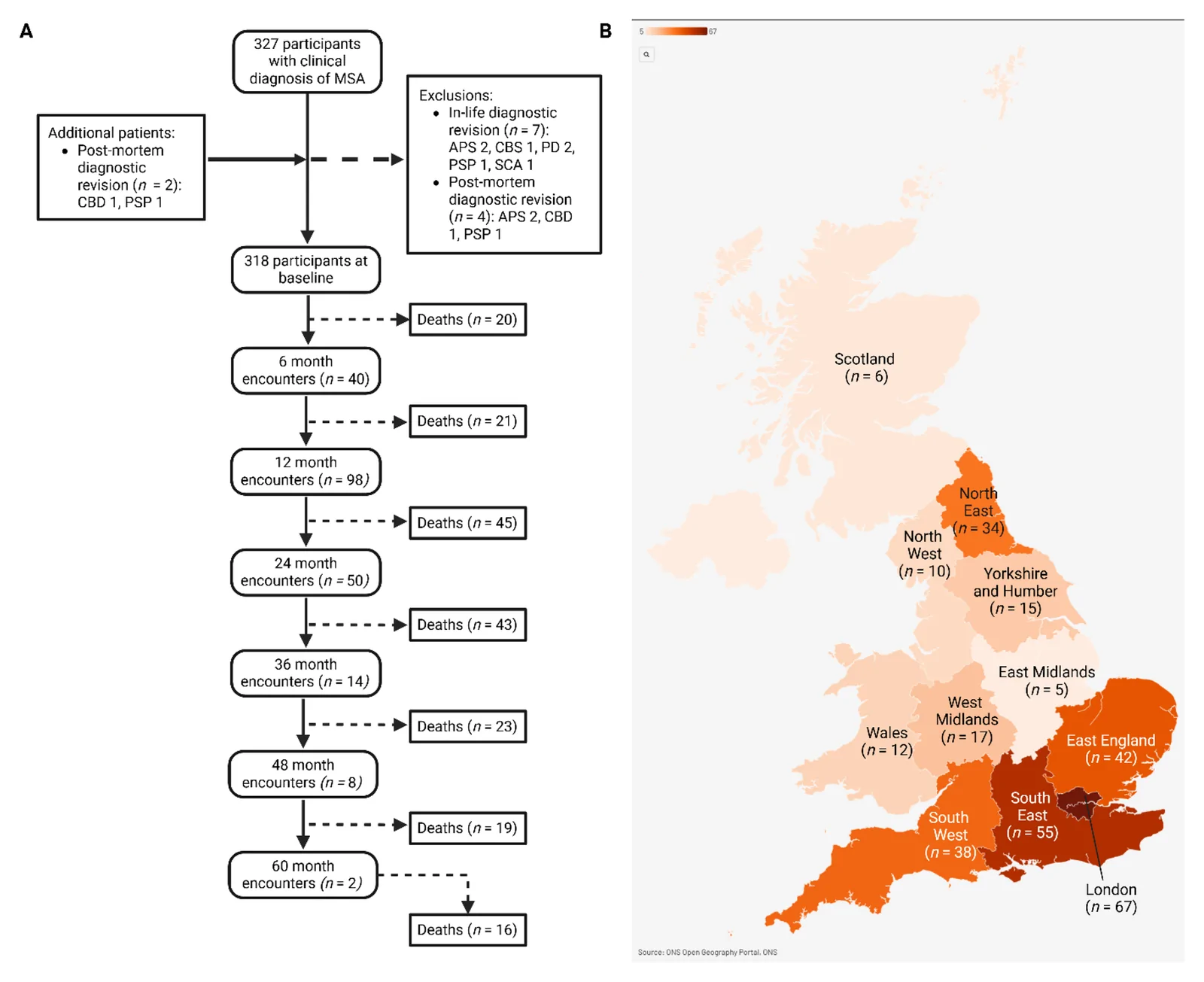
PROSPECT-M-UK participation (A) Flow diagram of MSA participant inclusion in PROSPECT-M-UK (B) Geographical distribution of PROSPECT-M-UK MSA participants.

Fig. 1A shows a participant inclusion flow diagram for the PROSPECT-M-UK participants, whilst Fig. 1B shows the geographical spread of participants where available.

Standardised instruments completed at each encounter can be found in Supplementary Table 2. Mean participant age at baseline encounter was 64.80 ± 8.30 years, whilst mean age of diagnosis was 62.9 ± 8.4 years. Median disease duration at diagnosis was 3.06 years (IQR: 1.92 - 4.92), whilst median disease duration at baseline encounter was 4.73 years (IQR: 3.31 - 6.63). 179 participants were followed up longitudinally, with a median follow up time of 2.22 years (IQR: 1.44 – 4.22 years).

20 PROSPECT-M-UK participants received neuropathological confirmation of MSA on post-mortem examination, 17 of whom were through the QSBB. For deceased PROSPECT participants (n=188), median survival from baseline visit was 2.11 years (IQR: 1.23 – 3.435), whilst median survival from disease onset was 7.78 years (IQR: 5.82-10.0).

Mean UMSARS ± SD at baseline visit was 45.1 ± 14.2. Mean UMSAR scores of the cohort at subsequent encounters can be seen in Supplementary Table 3, whilst other rating scores are in Supplementary Table 4.

### Disease milestone cohort comparison

Although the PROSPECT cohort reached most mobility related disease milestones (recurrent falls, walking aid use, outdoor wheelchair use) slightly earlier than the QSBB cohort, time between mobility milestones were similar between the cohorts, suggesting equivalent progression from point of recurrent falls (see Table 2). Progression to bulbar milestones was similar between the cohorts.

Although time to death from milestone attainment was later in the PROSPECT-M cohort than the QSBB cohort, the difference was not statistically significant. Importantly, in the combined cohort, median survival following use of an indoor wheelchair, gastrostomy insertion, or development of unintelligible speech was under 1.5 years, with upper bounds of the 95% confidence intervals for each milestone not exceeding 2.4 years, suggesting that these are relatively consistent late-stage disease markers.

### Survival from onset analyses

#### Effect of sex, onset symptom type and MSA clinical phenotype

In the combined cohort, there was no significant survival difference seen when comparing between sexes (*P* = 0.67), clinical phenotype (*P =* 0.78) and onset symptom type (*P* = 0.07) (See Supplementary Fig. 1). Unlike previous studies, initial presentation with dysautonomia did not affect survival from onset. Due to asymmetrical data collection on sexual dysfunction by sex, sensitivity analyses were performed, excluding erectile dysfunction as an onset symptom. This showed no change to the effect of sex (*P =* 0.68*)* or onset symptom type (*P =* 0.067) on survival.

### Effect of neuropathological subtype

**Figure 2:**
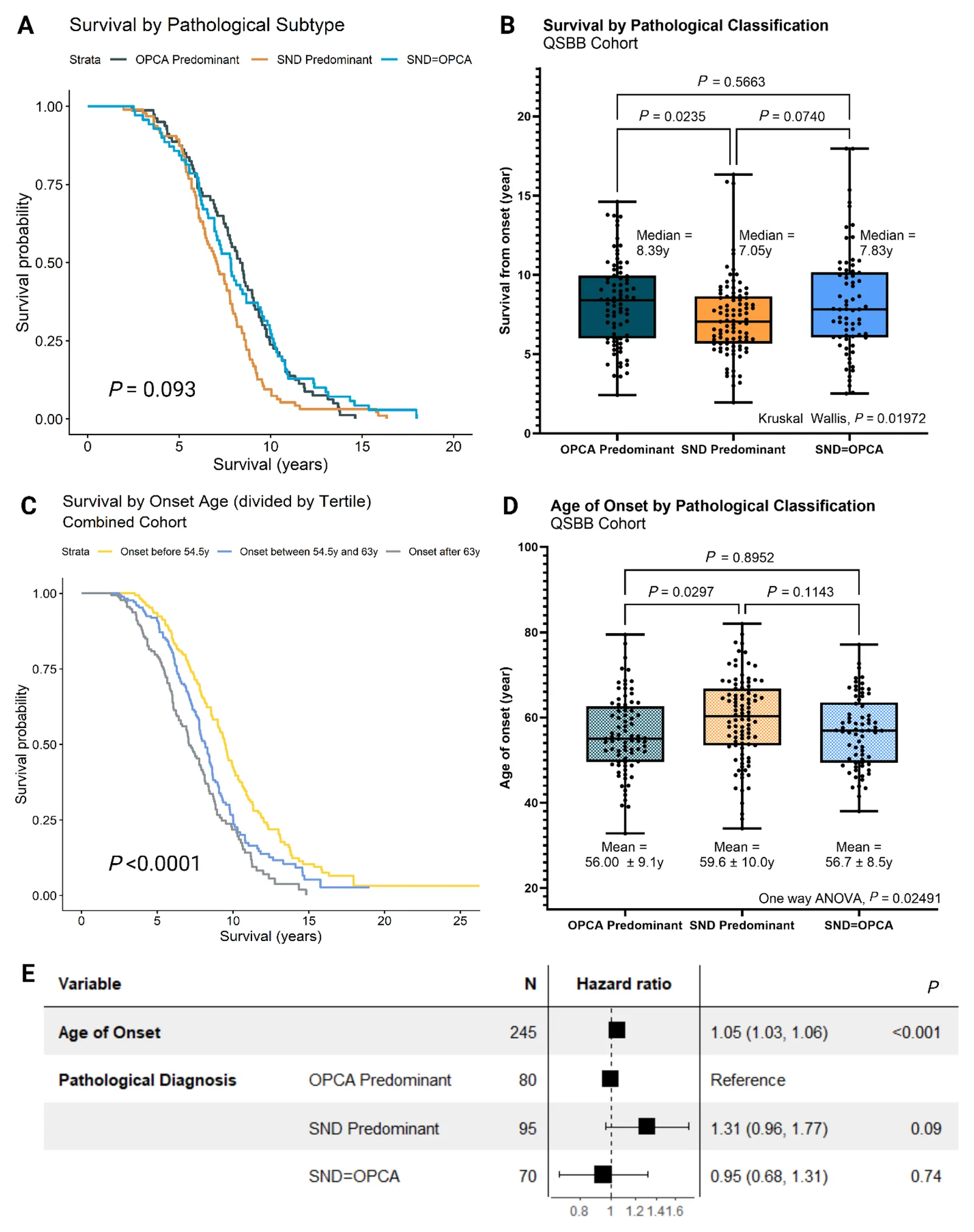
Effect of and Interaction between Onset Age and Pathological Classification on Survival from onset (**A**) Kaplan-Meier curve showing log rank p-value comparing between pathological subtypes (**B**) Dot-Boxplot illustration of pathological subtype group means (**C**) Kaplan-Meier curve comparing effect of age of onset with survival. **(D)** Box plot showing higher age at onset in SND compared to OPCA or mixed MSA patients. **(E)** Forest plot showing that the association between pathological subtype and reduced survival was attenuated after adjustment for age of onset.

In the QSBB neuropathological cohort, SND predominant MSA patients survived a shorter time from onset than OPCA predominant and SND=OPCA MSA patients (log-rank *P* = 0.0095, see Fig 2) with a hazard ratio (HR) of 1.46 (95% CI of 1.09 to 1.90, *P* = 0.0129). There were insufficient datapoints for a similar PROSPECT-M-UK pathologically confirmed cohort comparison.

### Effect of age of onset and diagnosis

Later disease onset was associated with shorter survival from onset in both cohorts, with a HR of 1.05 (95% CI: 1.03-1.07, *P* < 0.0001) for the QSBB cohort and 1.04 (95% CI: 1.03-1.06, *P* < 0.0001) for the PROSPECT cohort.

Age of onset was later in participants with SND-predominant MSA (59.6 ± 10.0y) compared to OPCA-predominant (56.0 ± 9.1y) and SND=OPCA (56.7 ± 8.5y), *P =* 0.025 (See Fig. 2). When included in a multi-variable Cox regression analysis with age of onset, effect of pathological subtype was lost, whilst age of onset remained significant. No interaction terms nor markers of collinearity were significant on analysis.

Time from symptom onset to diagnosis was available in 282 PROSPECT cohort participants. When included in a Cox proportional hazards analysis, shorter time to diagnosis was associated with shorter survival from onset even after accounting for age of onset (HR = 0.86, 95% CI (0.80-0.92), *P* < 0.001). There were no significant interactions between co-variates.

### Mobility and bulbar milestones in the combined cohort

#### Cox proportional hazards time-dependent co-variate analysis

Time-dependent co-variate Cox analysis showed that early development of all disease mobility and bulbar milestones examined was associated with shorter survival (see Table 3). For example, after adjusting for age of onset, a patient who develops recurrent falls, has 2.11 times the hazard of mortality as those who do not (HR 2.11, 95% CI = 1.5-2.97, *P* < 0.0001).

Combining the QSBB and PROSPECT cohorts, multivariable Cox proportional hazards analysis was performed using only covariates available in both cohorts with stepwise selection. Variance influence factors (VIF) were reviewed, and time to outdoor wheelchair use and gastrostomy insertion were excluded due to multi-collinearity and event number requirements of >15 per covariate.

The final multivariable model identified that recurrent falls (HR 1.87, 95% CI 1.32-2.66, *P* < 0.001), indoor wheelchair use (HR 1.99, 95% CI 1.42-2.78, *P* < 0.001) and unintelligible speech (HR 1.61, 95% CI 1.13-2.29, *P* = 0.008) were associated with shorter survival from onset. No interactions terms were significant in this model. Sensitivity analysis excluding erectile dysfunction as an onset symptom did not result in any change of significance of this result.

Univariate Cox analysis looking at the effect of time to milestone development on survival in the separate QSBB and PROSPECT cohorts showed comparable results across the two cohorts (see Supplementary Table 5). In both cohorts, earlier attainment of disease milestones such as walking aid and wheelchair use, gastrostomy insertion and unintelligible speech, was associated with shorter survival.

#### Landmark analysis at 3, 4 and 5 years from symptom onset

Univariable analysis at the 3 separate time points, adjusted for age of onset and multiple testing is shown in Table 4. At 3 years from symptom onset, patients with recurrent falls (HR 1.57, 95% CI: 1.15-2.13, *P* = 0.004) and unintelligible speech (HR 3.29, 95% CI: 1.23-8.80, *P* = 0.018) had significantly higher hazard of mortality than those without. Walking aid use trended toward significance at 4 years (HR 1.48, 95% CI: 0.96-2.29, *P* = 0.075), whilst outdoor and indoor wheelchair use became significant at the 5-year landmark.

To allow more convenient clinical translation, we investigated 10-year survival rates from symptom onset using the landmark survival analyses cohort subsets.

**Figure 3:**
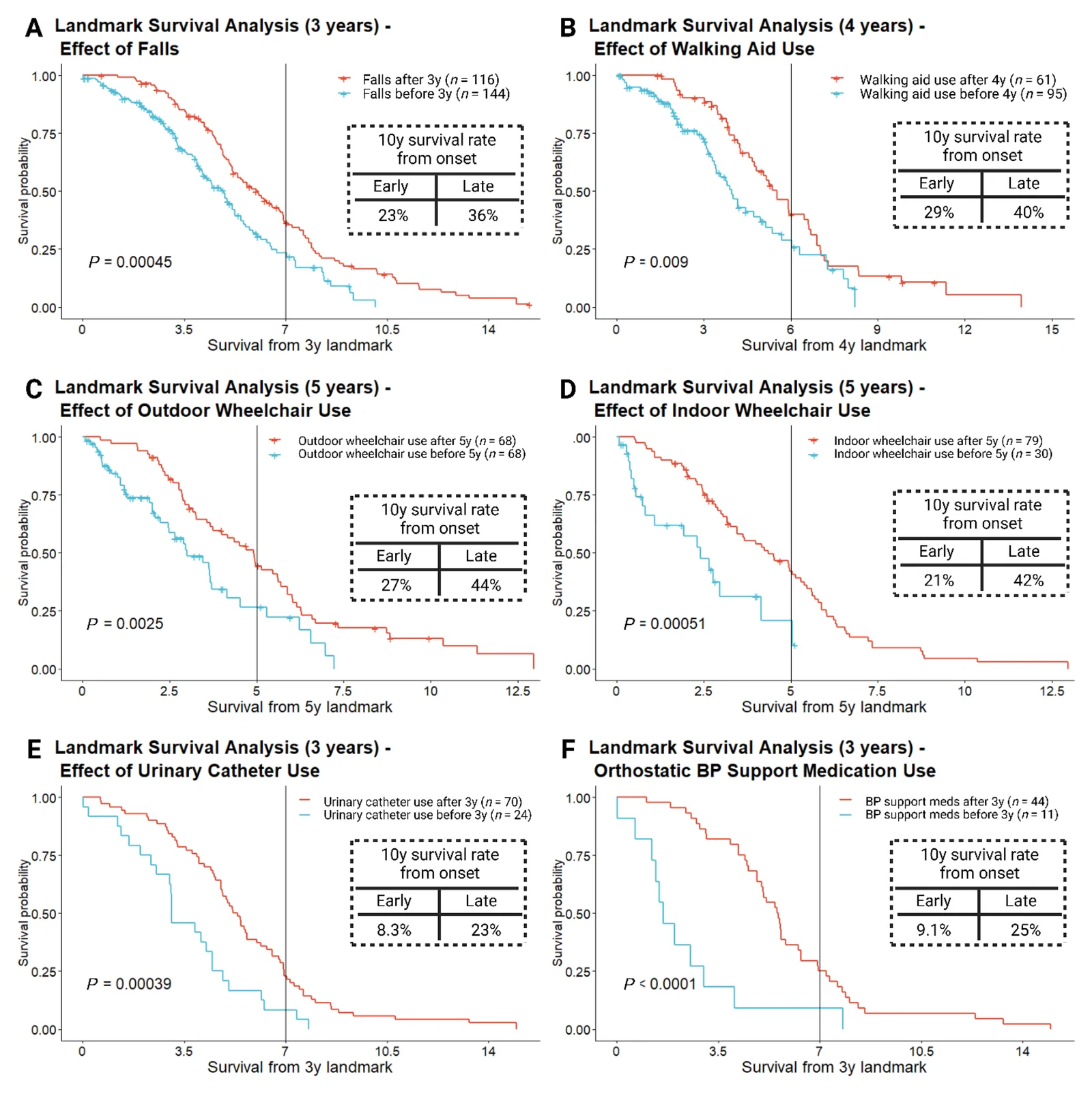
Landmark Analysis Survival Curves showing that disease milestone attainment can predict prognosis. Vertical lines in each graph illustrate survival separation at 10 years for the different groups. Early vs Late refers to milestone attainment as defined by presence of disease milestone at point of landmark analysis.

Fig. 3A-D shows the Kaplan Meier curves for the landmark analyses and provides more specific 10-year survival rates to contextualise these results. For example, patients experiencing recurrent falls by Year 3 from disease onset, 10-year survival was 23% compared to 36% for those without (log rank *P* = 0.001). Indoor wheelchair use by Year 5 is associated with a 10-year survival of 21% compared to 42% otherwise (*P* < 0.001).

#### Gastrostomy insertion

Given the findings that respiratory tract infection was a common cause of death in MSA patients, we explored the dataset to see if gastrostomy insertion was associated with subsequent longer survival as it may affect aspiration risk. In the combined cohort, 85 patients were recommended gastrostomy at some stage. 52 of these had gastrostomies inserted whilst 33 remained without. Reasons for non-insertion were not documented. There was no difference in survival between the two groups (log rank *P* = 0.68), with mean survival of 8.7 ± 3.0 years vs 8.1 ± 2.9 years (inserted vs not).

57 of the 85 patients had documented first gastrostomy recommendation dates. Subgroup analysis of this smaller cohort showed that mean survival from gastrostomy recommendation was longer in those with gastrostomies inserted (n = 24) at 2.1 ± 1.7 years vs 1.2 ± 1.4 years where no gastrostomy was inserted (n = 33), however, this did not reach significance (log rank *P* = 0.08).

### Dysautonomia milestones – QSBB cohort only

Dysautonomia associated milestones were only available for the QSBB cohort. Multi-variable Cox analysis showed that early catheter insertion (HR 0.67, 95% CI: 0.51-0.89, *P* = 0.006) and use of orthostatic medications (HR 0.46, 95% CI: 0.31-0.69, *P* < 0.001) were significantly associated with shorter survival from onset (See Supplementary table 6), but not early urinary incontinence (HR 0.89, 95% CI: 0.63-1.25, *P* = 0.49). Due to low event numbers, combined modelling of the dysautonomia and mobility associated milestones could not be performed.

#### Landmark analysis at 3, 4 and 5 years from symptom onset

Landmark analysis using dysautonomia milestones showed that at 3 years from onset, needing a urinary catheter (log rank *P* < 0.001) and regular orthostatic medication use (log rank *P*<0.001) are negative prognostic markers (See Fig. 3E-F).

By 5 years, however, the effect of both had diminished, with non-significant impact on survival, although catheter use continued to trend towards a negative survival outcome (See Table 4).

### Standardised rating scales – PROSPECT-M-UK cohort only

#### UMSARS and MSA QoL

**Figure 4:**
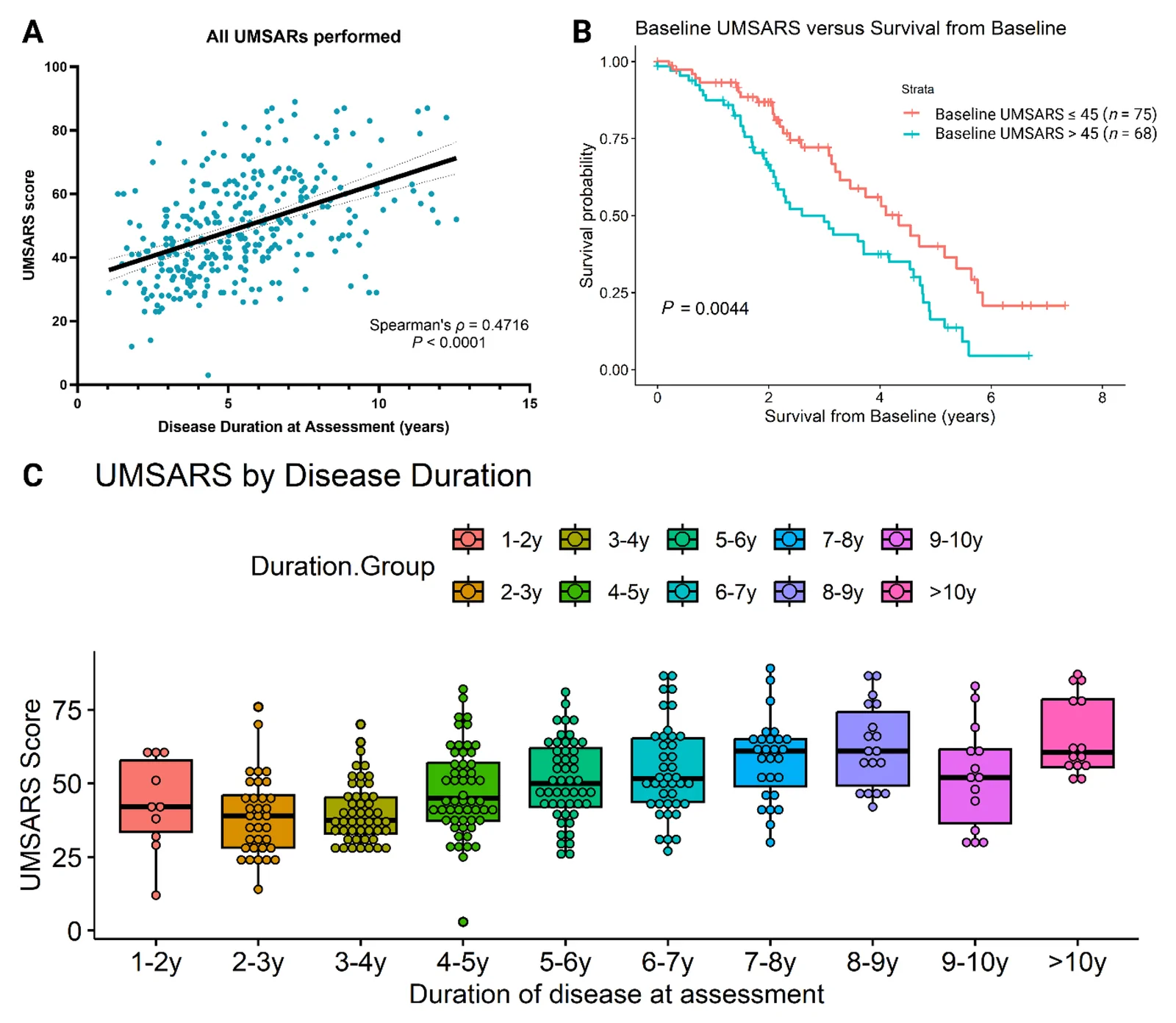
Association between UMSAR scores, disease duration and prognosis. (**A and C**) UMSARS scores correlated with disease duration at time of assessment, however at may plateau after 7 years (**B**) A baseline cut-off UMSARS score of 45 showed differences in survival outcomes.

Mean UMSARS ± SD at baseline was 45.10 ± 14.20 with mean UMSARS progression over time previously shown in Table 1. Longer disease duration at assessment was associated with higher UMSARS score (R=0.472, *P* < 0.001), although this effect appears to plateau after 7 years (See Fig. 4C).

Higher baseline UMSARS scores were associated with shorter survival from baseline assessment, (HR 1.026, 95%CI: 1.009-1.042, *P* = 0.002), but did not correlate with survival from onset (HR 0.9996, 95%CI: 0.984-1.016, *P* = 0.962). A baseline UMSARS score of >45 was associated with shorter survival from baseline assessment.

##### Rate of change of UMSARS

Simple linear regression using 91 patients with multiple longitudinal UMSARS scores, gave each an UMSARS progression rate. Median rate of UMSARS change per year was 10.27 points (IQR 5.31-14.27). There was no significant correlation between rate of change of UMSARS and baseline UMSARS score (R=-0.41, *P =* 0.41) (See Fig. 4A). Higher rate of UMSARS change from baseline was associated with shorter survival from baseline assessment (HR= 1.065, 95%CI: 1.034-1.098, *P <* 0.001)

##### Baseline MSAQoL scores

Although univariable analysis showed that baseline MSAQoL scores were associated with shorter survival from baseline assessment (HR 1.012, 95% CI: 1.006-1.019, *P <* 0.001), when factored into a multivariable analysis considering age of onset, total UMSARS and rate of change of UMSARS, this association was no longer significant.

## Discussion

### Impact of findings on clinical prognostication in MSA

With 555 patients, this is the largest study of neuropathologically confirmed and prospectively collected MSA patients to date, which has allowed us to gather unique insights into MSA disease progression and prognosis.

In our study, we focused on prognostic factors that could be identified through clinical history or examination, with the aim of providing clinically useful predictors.

There are three key findings from this work that we would like to highlight;

Firstly, our data suggests that disease prognostic factors vary in the mortality risk they pose at different disease stages.

At 3 years from disease onset, landmark analysis showed that factors such as falls, unintelligible speech, catheter use and need for blood pressure medication were associated with shorter survival. From that point, however, the significance of autonomic milestones waned, whilst mobility milestones increased mortality risk. For example, a patient who requires a catheter within their first 2 years of disease onset is at high risk of early mortality, but once the same patient reaches 5 years from onset, their risk of mortality is similar to patients without catheters.

This finding may explain previous disparities in similar cohort studies and should be considered when performing future research in this area. This could also affect clinical trial analysis and the establishment of inclusion and exclusion criteria, potentially allowing a larger pool of eligible patients.

Secondly, our study identified markers of end-stage disease such as onset of indoor wheelchair use, gastrostomy insertion and unintelligible speech where median survival from milestone occurrence was less than 1.5 years with a 95% confidence interval upper limit of 2.4 years at latest. These results are of clinical relevance when considering advanced care planning discussions and explaining prognosis to patients.

This data must be considered in context, however, as timing of interventions such as wheelchair use and gastrostomy insertion differs between individuals depending on personal circumstances and preference. We would also caution that these findings do not indicate that delayed intervention will prolong life, as our data was not equipped to study this outcome.

Third, our results suggest that in clinic UMSARS assessment can support prognostication, both as a single mid-disease timepoint assessment as well as when considering rate of change over time.

### Cause of death in MSA

In cases where the cause was known, respiratory infections were the most common cause, representing 66% of deaths. This is unsurprising, given the dysphagia, difficulty with nutritional intake and immobility MSA patients suffer. Pneumonia as a terminal event is well-recognized in other atypical parkinsonian syndromes and was the cause of death in 40.9-45% of patients with progressive supranuclear palsy^24,25^. The comparatively high proportion affected in our cohort could be due to more severe dysphagia in MSA with bulbar cerebellar incoordination or could be a result of study size differences. In addition, cause of death was only available in 56.5% of study participants, which could further affect these results.

In patients where gastrostomy was recommended in our combined cohort, insertion did not significantly affect survival outcomes. Interestingly, this contrasted with a previous 2024 study reporting on the longitudinal and natural history arms of the PROSPECT-M-UK cohort of atypical parkinsonism patients. This showed significantly longer survival in atypical parkinsonism patients with who received gastrostomies compared to those where it was recommended but not inserted^26^. However, this could be due to cohort size, where of the 44 patients where gastrostomy was recommended in that study, only 13 had MSA whilst our cohort here reports on 85 MSA patients known to have gastrostomies recommended.

In our cohort, reasons for non-insertion were not documented and may include increased baseline frailty or other co-morbidities that could affect survival. We also did not collect information on advance care plans and potential refusal of intravenous antibiotics or hospital admission. Also crucial to the question of gastrostomy, our data was not equipped to study its impact on patients’ quality of life – a consideration of utmost importance to those affected. As such, we cannot draw any conclusions around gastrostomy effectiveness, however, note that more research in this area is needed.

### Baseline comparison within cohort and with previous studies

In our combined cohort, men and women were equally affected, mean age of onset was 58 years with a predominance of MSA-P, in keeping with other previously reported Caucasian cohorts.

Median survival in the QSBB cohort was shorter than the PROSPECT cohort at 7.62 years vs 9.11 years, despite an earlier age of onset. However, the PROSPECT cohort had comparable median survival (as calculated with Kaplan-Meier curve fitting) to previously published prospective natural history studies (see Table 8).^4,7,8^ Other neuropathological studies have reported median survival of between 7.9-8.6 years, not dissimilar to our neuropathological cohort.^12,14^ This difference could reflect referral bias as younger patients with faster disease progression (i.e. ‘more atypical’ cases) may be more likely to be referred for neuropathological evaluation. Further, median time to baseline assessment of the prospective cohort was 4.73 years, which could lead to guarantee time bias, where those with shorter survival may not have been included.

Further comparison with other natural history studies showed similar disease durations and ages at baseline assessment.

The PROSPECT cohort’s mean baseline UMSARS was 45.1 points, increasing by an average of 15 points over 2 years. Our patients’ UMSARS were lower and slower to progress than the EMSA cohort (baseline 51.2), increasing by 18 points to 69.5 after 2 years.^8^ Our patients progressed faster than the North American cohort however (only 8.8 points over 2 years) and reported a higher baseline UMSARS than the French Reference Centre cohort’s 40.8 points (See Supplementary Table 8 for a more detailed comparison).^4,7^

### Study limitations and mitigations

The large number of neuropathologically confirmed MSA patients in our cohort has been useful in avoiding inaccuracies arising from clinical misdiagnosis in life.

However, despite its size, we acknowledge our study’s limitations.

Firstly, the post-mortem cohort data collection was retrospective in nature, and therefore subject to issues with missing data and recall bias. To minimize this, data collection was performed using a pre-defined data dictionary, limiting onset to core diagnostic criteria, excluding constipation and REM sleep behaviour disorder.

In addition, strict error margins (of ±2 months) were applied to onset timing to increase accuracy. However, this has led to reduced data points and low event numbers available for survival analysis. This is evident, for example in the unusually low number of patients where timing of first catheterization was available, only 100 from a cohort of 254.

With missing data here, imputation was not possible as the assumption of randomness could not be made. With this, we have elected to use complete case analysis, and where possible, have employed sensitivity analyses to test the robustness of the results reported. For several prognostic factors studied, we were only able to apply univariable regression analysis, due to low event numbers, making a full analytical model difficult to estimate.

## Conclusion

To our knowledge, this is the largest combined post-mortem and prospective study reviewing clinical prognostic factors in MSA. From this, we conclude that clinically available rating scales and historical prognostic markers can be used to predict survival from onset as well as survival from time of assessment. Importantly, some clinical prognostic markers are time dependent, affecting mortality risk differently over the course of the disease.

This knowledge can support clinicians when counselling patients on prognosis and guide future clinical trial design through the refinement of inclusion and exclusion criteria, allowing for better patient stratification.

## Supplementary Material

Supplementary material is available at *Brain* online.

## Figures and Tables

**Table 1:** Patient Cohort Demographics

	QSBB Post-mortem cohort	PROSPECT-M-UK cohort	Combined cohort (duplicates removed)
Total number of participants	254	318	555
Male : Female	130 : 124	186 : 132	306 : 249
Mean age of onset (y)	57.58 ± 9.34	59.45 ± 8.88	58.65 ± 8.99
Mean age of death (y)	65.37 ± 8.62	67.88 ± 8.32	66.4 ± 8.44
Median Survival from onset (y) (95% CI)^a^	7.63 (7.09–7.95)	9.11 (8.61–9.88)	8.25 (7.88–8.63)
**Neuropathological sub-classification**			
SND Predominant	110	4^b^	110
OPCA Predominant	83	8^b^	83
Mixed SND/OPCA	74	4^b^	74
Minimal change	5	1^b^	5
Unspecified	6	4^b^	10
**Clinical phenotype sub-classification**			
MSA-P	55	111	160
MSA-C	57	83	132
MSA – Phenotype not specified	104	120	223
PD^c^	18	-	18
APS^c^	2	2	3
CBS^c^	2	1	2
PSP^c^	12	1	12
PAF^c^	1	-	1
Not documented^c^	3	0	3
**Ethnicity**			
White	106	253	349
White-Mixed	0	4	4
Black	1	2	3
Asian – Indian	1	7	8
Asian - Pakistani	0	3	3
Asian - Chinese	0	5	5
Asian – Other	0	3	3
Not documented	146	41	180
**Survival (deceased pts only)**			
<5y	37 (14.6%)	32 (17.0%)	65 (15.4%)
5-12y	198 (78.0%)	1 24 (70%)	306 (72.7%)
>12y	19 (7.5%)	19 (10.1%)	37 (8.8%)
Not documented	NA	1 3 (6.9%)	13 (3.1%)

PD = Parkinson’s Disease, APS = Atypical Parkinsonian Syndrome, CBS = Corticobasal Syndrome, PSP = Progressive Supranuclear P alsy.

a Kaplan-Meier curve estimated median survival used for PROSPECT and combined cohorts.

b Seventeen participants were enrolled in both cohorts, and duplicates have been removed from the combined cohort.

c Diagnoses made in-life prior to post-mortem confirmation of MSA.

**Table 2:** Median time to disease milestones in both cohorts where event date is known

Milestone	QSBB Cohort		PROSPECT-M-UK Cohort		Combined Cohort		Wilcox *(W)*	Adjusted *P*-values^a^
	n	Median (IQR)	n	Median (IQR)	n	Median (IQR)		
**Time to Milestones**								
Recurrent falls	146	3.04 (1.60–5.00)	127	1.92 (0.50–3.46)	268	2.61 (1.00–4.87)	6236	**0.000066**
Walking aid use	61	4.00 (2.54–6.00)	121	3.00 (1.92–4.39)	179	3.20 (2.00–4.80)	2697	**0.0176**
Outdoor wheelchair use	72	5.14 (4.41– 6.92)	84	4.09 (3.1 1–6.47)	155	4.75 (3.71–6.57)	2067	**0.0048**
Indoor wheelchair use	77	6.00 (4.99– 7.92)	44	5.20 (3.98–6.94)	121	5.77 (4.45–7.64)	1254	0.0555
Gastrostomy insertion	41	7.00 (5.95– 8.12)	11	6.95 (4.99–7.81)	52	6.98 (5.72–8.13)	194	0.696
Gastrostomy recommendation	33	6.33 (5.46– 7.83)	30	6.50 (5.01–8.29)	57	6.46 (5.41 –7.92)	352	0.677
Unintelligible speech	50	6.17 (5.22– 7.97)	30	6.37 (4.65-9.19)	79	6.17 (5.12-8.95)	759	0.72
Catheter use	96	4.37 (2.89– 6.43)	–	–	–	–	–	–
Urinary incontinence	75	2.00 (1.00– 3.84)	–	–	–	–	–	–
Use of orthostatic medications	56	4.15 (3.00– 6.83)	–	–	–	–	–	–
**Time between mobility milestones**								
Falls to walking aid use	26	1.19 (0.67– 1.67)	79	1.41 (0.83–2.47)	103	1.33 (0.71–2.40)	3268	0.29
Walking aid to outdoor wheelchair use	34	1.37 (0.73– 2.00)	69	1.38 (0.67–2.00)	102	1.36 (0.71–2.00)	1338	0.89
Outdoor to indoor wheelchair use	43	1.08 (0.63– 1.63)	31	0.928 (0.50–1.31)	74	1.00 (0.58–1.56)	940	0.33
**Time to death from milestones**								
Falls to death	146	3.91 (2.48– 6.04)	54	4.52 (2.94–6.18)	193	4.04 (2.66–6.09)	3966	0.66
Walking aid use to death	61	3.46 (2.23– 4.17)	53	3.40 (2.05–5.17)	111	3.49 (2.15–4.68)	1648	1.00
Outdoor wheelchair use to death	72	2.01 (1.09– 3.13)	40	2.37 (1.48–3.62)	109	2.18 (1.48–3.30)	1558	0.54
Indoor wheelchair use to death	77	1.39 (0.78– 2.32)	23	1.97 (1.22–3.17)	99	1.46 (0.91–2.43)	1079	0.30
Gastrostomy insertion to death	41	1.02 (0.26– 1.87)	6	2.03 (1.32–3.24)	47	1.06 (0.31–2.05)	182	0.36
Gastrostomy recommendation	33	0.72 (0.24– 1.54)	19	0.95 (0.59–1.91)	44	0.90 (0.53–2.08)	258	0.10
Unintelligible speech to death	50	1.17 (0.55– 2.35)	20	1.86 (1.03–2.50)	68	1.54 (0.73–2.43)	571	0.56

IQR = Interquartile Range.

a *P*-values compare between the QSBB and PROSPECT cohorts using the Wilcoxon test for non-parametric data and have been adjusted for multiple testing. Adjusted P-values of <0.05 are in bold.

**Table 3:** Cox Analysis of Time Dependent Covariates^a^

Time Dependent Covariate	Median Years (IQR)	Combined Cohort Individual Covariate Analyses			Combined Cohort Multivariable Cox Analysis (n=48)	
		*n*	HR (95% CI)	Adjusted p-value	HR (95% CI)	Adjusted p-value
Recurrent falls	2.61 (1.00–4.87)	268	2.11 (1.50–2.97)	**1.89 × 10^–5^**	1.87 (1.32–2.66)	**0.000477**
Walking aid use	3.20 (2.00–4.80)	179	1.34 (1.03–1.74)	**0.0308**	0.86 (0.65–1.14)	0.291
Outdoor wheelchair use	4.75 (3.75–6.57)	155	1.78 (1.37–2.33)	**2.09 × 10^–5^**	NA	NA
Indoor wheelchair use	5.77 (4.45–7.64)	121	2.78 (2.11 –3.65)	**3.18 × 10^–13^**	1.99 (1.42–2.78)	**5.47 × 10^–5^**
Gastrostomy insertion	6.98 (5.72–8.13)	52	2.95 (2.09–4.17)	**9.53 × 10^–10^**	NA	NA
Unintelligible speech	6.17 (5.12–8.95)	79	2.53 (1.87–3.42)	**1.85 × 10^–9^**	1.61 (1.13–2.29)	**0.008482**
Final age of onset	58.5 (52.2–65.1)	534	1.0 (1.0– 1.1)	**4.3 × 10^–10^**	1.03 (1.01–1.05)	**0.00158**

NA = Not applicable. Time to outdoor wheelchair use and gastrostomy insertion were excluded from the multivariable analysis d ue to multi-collinearity and event number requirements of >15 per covariate

a Results adjusted for both age of onset and multiple testing. Results in bold indicate P-values of <0.05.

**Table 4:** Cox analysis at different landmark survival analysis stages

	3 years		4 years		5 years	
	Adjusted HR (95% CI)	Adjusted *P* value^a^	Adjusted HR (95% CI)	Adjusted *P* value^a^	Adjusted HR (95% CI)	Adjusted *P* value^a^
Falls	**1.57 (1.15–2.13)**	**0.004**	**1.86 (1.35–** **2.58)**	**<0.001**	**1.98 (1.39–** **2.82)**	**<0.001**
Walking aid use	0.84 (0.55–1.29)	0.44	**1.48 (0.96–** **2.29)**	0.075	**1.70 (1.06–** **2.74)**	**0.029**
Outdoor wheelchair use	0.83 (0.41–1.68)	0.61	0.78 (0.46–1.34)	0.37	**1.93 (1.25–** **2.97)**	**0.003**
Indoor wheelchair use	1.92 (0.70–5.35)	0.20	1.52 (0.65–3.55)	0.33	**2.62 (1.50–** **4.62)**	**<0.001**
Gastrostomy insertion	NA^b^	NA	NA^b^	NA	2.02 (0.76–5.33)	0.158
Unintelligible speech	**3.29 (1.23–8.80)**	**0.018**	**3.87 (1.54–9.75)**	**0.004**	**3.10 (1.34–** **7.15)**	**0.008**
Catheter use	**1.76 (1.03–3.00)**	**0.038**	**1.87 (1.16–** **2.99)**	**0.001**	1.62 (0.98–2.68)	0.0601
Orthostatic medication	**3.29 (1.44–7.54)**	**0.005**	1.79 (0.86–1.05)	0.125	1.53 (0.78–2.99)	0.212

CI = Confidence Interval; HR = Hazard Ratio.

a All results adjusted for age of onset and multiple testing. Results in bold indicate P-values of <0.05.

b NA = Not applicable. At 3 and 4 years, the PEG insertion group only had 2 patients for analysis, and so the results were excluded.

## Data Availability

The synthesised data supporting the findings of this study are available on reasonable request to the corresponding author. Raw data are not publicly available as they contain information that could compromise research participant privacy.
